# Novel Anion-Exchange Resins for the Effective Recovery of Re(VII) from Simulated By-Products of Cu-Mo Ore Processing

**DOI:** 10.3390/ijms26157563

**Published:** 2025-08-05

**Authors:** Piotr Cyganowski, Pawel Pohl, Szymon Pawlik, Dorota Jermakowicz-Bartkowiak

**Affiliations:** 1 Department of Process Engineering and Technology of Polymer and Carbon Materials, Wroclaw University of Science and Technology, 27 Wybrzeze St. Wyspianskiego, 50-370 Wroclaw, Poland; spawlik98@gmail.com (S.P.); dorota.jermakowicz-bartkowiak@pwr.edu.pl (D.J.-B.); 2 Department of Analytical Chemistry and Chemical Metallurgy, Wroclaw University of Science and Technology, 27 Wybrzeze St. Wyspianskiego, 50-370 Wroclaw, Poland; pawel.pohl@pwr.edu.pl

**Keywords:** ion exchange, functional resins, hydrometallurgy, metal recovery

## Abstract

The efficient recovery of rhenium (Re), a critical metal in high-tech industries, is essential to address its growing demand and reduce reliance on primary mining. In this study, we developed novel anion-exchange resins for the selective adsorption and recovery of Re(VII) ions from acidic solutions, simulating industrial by-products. The resins were synthesized from a vinylbenzyl chloride-co-divinylbenzene copolymer modified with aliphatic, heterocyclic, and aromatic weakly basic amines, selected from among bis(3-aminopropyl)amine (BAPA), 1-(2-pyrimidinyl)piperazine (PIP), thiosemicarbazide (TSC), 2-amino-3-hydroxypyridine (AHP), 1-(2-hydroxyethyl)piperazine (HEP), 4-amino-2,6-dihydroxypyrimidine (AHPI), and 2-thiazolamine (TA). The adsorption of Re on BAPA, PIP, and HEP resins obeyed the Langmuir model, and the resins exhibited high adsorption capacities, with maximum values reaching 435.4 mg Re g^−1^ at pH 6. Furthermore, strong selectivity for ReO_4_^−^ ions over competing species, including Mo, Cu, and V, was noted in solutions simulating the leachates of the by-products of Cu-Mo ores. Additionally, complete elution of Re was possible. The developed resins turned out to be highly suitable for the continuous-flow-mode adsorption of ReO_4_^−^, revealing outstanding adsorption capacities before reaching column breakthrough. In this context, the novel anion-exchange resins developed offer a reference for further Re recovery strategies.

## 1. Introduction

Rhenium (Re) is a rare and precious metal known for its unique properties, such as its high melting point, excellent thermal stability, and resistance to corrosion [1,2]. Because of these properties, Re species have been applied in many industrial and technological fields, including metallurgy, petrochemistry, medicine, and even the aerospace and nuclear sectors [2,3,4]. These applications include the use of Re as a component of superalloys intended for use in the production of gas turbine engine blades, coatings for face-seal rotors, jet engines, and electrodes [1]. Furthermore, Re is used as a catalyst for reforming naphtha in gasoline or for producing petroleum refinement catalysts enabling increases in the octane number of gasolines [5]. All of these applications make Re indispensable in a variety of industrial applications, resulting in a constantly growing demand [6,7,8,9]. The growing demand for Re in high-tech industries has made its recovery from secondary sources, such as by-products of copper–molybdenum ore processing [7,10,11,12,13,14,15,16,17,18] or wasted superalloys and catalysts [19,20,21,22,23,24], an increasingly important research topic. The above-mentioned by-products often contain significant amounts of Re in the form of Re(VII) ions [14]; however, it can be challenging to recover them efficiently. Current technologies are hindered by the complex nature of the secondary sources’ matrices and the presence of competing ions such as copper (Cu), molybdenum (Mo), vanadium (V), and tungsten (W) within them [10,12,13,19,21,25,26,27]. The development of novel materials and methods for the selective recovery of Re from such sources is therefore crucial for both environmental sustainability and efficient use of this valuable metal [7,21,23,28].

The scarcity of Re and its critical importance in industrial applications, particularly when used as a catalyst in the petroleum and chemical industries [8,9,29,30], underscore the necessity of developing effective recovery methods. In its natural state, Re is typically found in low concentrations in ores, often associated with other metals such as Mo [6,10,31]. Traditional methods for Re extraction, including hydrometallurgical and pyrometallurgical processes, often fall short in terms of their efficiency, selectivity, and environmental sustainability [21,23,28]. In the processing of Cu-Mo ores, Re typically occurs as a trace element in molybdenite (MoS_2_) and is released during the oxidative roasting or leaching steps, primarily in the form of perrhenate ions (ReO_4_^−^). The resulting process solutions often contain complex acidic mixtures rich in Cu, Mo, and other metals, with Re present at low concentrations. These solutions pose a significant challenge for selective Re recovery due to the competing interactions from coexisting species such as MoO_4_^2−^, Cu^2+^, and VO^3−^ [32,33]. Furthermore, according to Liu et al. [23], these methods also tend to be energy-intensive and costly, making it essential to explore alternative techniques that can improve Re recovery [23]. In this context, anion-exchange resins are a promising solution to overcoming the above-mentioned inconveniences because of their high selectivity, ability to be easily regenerated, and suitability for use in continuous processes [21].

Anion-exchange resins, particularly those modified with specific functional groups, have been proven to be effective for the selective adsorption and recovery of various metals existing in anionic forms, including Re [4,16,24,34,35,36]. These resins work through the ion-exchange mechanism, where ReO_4_^−^ anionic species in the solution are exchanged for counter-ions on the resin surface. They are versatile, especially when tailored for the specific requirements of the target metal, and therefore increasingly used for the recovery of Re from both primary and secondary sources [21,37]. Among the various strategies explored, the modification of the resin surface with amine groups (aliphatic, heterocyclic, and aromatic) has been shown to provide promising results because it increases the resin’s affinity for ReO_4_^−^ ions [4,14,16,24,34,35,36,37,38,39]. These modifications can improve the resin’s capacity for Re(VII) uptake, increase its selectivity, and enhance its overall performance in real-world conditions. In this context, while anion-exchange resins have proven effective for rhenium recovery, existing commercial resins often suffer from limitations, such as low selectivity in the presence of competing anions (e.g., molybdate) and decreased efficiency in strongly acidic environments. For example, Fathi et al. [4] demonstrated that the Purolite A170 resin exhibits diminished selectivity in Re-Mo binary systems, while Liu et al. [34] noted that the performance of weak-base resins is highly dependent on pH and solution composition. Hou et al. [35] showed that modified D201 resins can achieve high Re(VII) adsorption from leachates, but their performance is also sensitive to competing ions.

Recent studies have focused on the synthesis of novel polymeric anion-exchange resins for the adsorption and recovery of Re from acidic solutions, simulating secondary sources, particularly those that arise during the processing of Cu-Mo ore [13,28,35,36,39]. The complexity of these solutions, which often contain high concentrations of co-existing metals, presents a challenge in terms of the selective recovery of ReO_4_^−^ ions. In this context, the incorporation of appropriate, N-rich functional groups into polymeric matrices facilitates a desirable increase in the selectivity of ReO_4_^−^ [36,37]. Indeed, amino-functionalized resins have been shown to exhibit enhanced selectivity and capacity for ReO_4_^−^ ions, allowing for the efficient separation of Re from other metal ions in the solution. Such resins can also be tailored to maximize their adsorption capacity and optimize operating conditions for Re recovery, thus improving the overall sustainability of this process [21,24].

Despite successful applications of ion-exchange techniques for the separation and recovery of Re from various sources, there is still a critical need to invent new and better ion-exchange materials capable of significantly improving Re recovery efficiency [4,40]. Furthermore, with the depletion of easily accessible sources of Re, this demand is expected to rise [16,23,24,37,38], highlighting the urgency of upgrading ion-exchange technologies to address these processing challenges.

To address these challenges, we introduce newly synthesized anion exchangers bearing aliphatic, heterocyclic, and aromatic amines. These resins were tailored to improve perrhenate selectivity, even when applied to complex multi-ionic mixtures representative of real secondary streams. It has been hypothesized that amino functionalities with a weakly basic (WB) characteristic and a “complex” molecular structure, particularly those containing heterocyclic or aromatic rings with various substituents, can enhance the selective uptake of ReO_4_^−^ ions over other competing species in multi-ionic systems [4,40]. This hypothesis is grounded in prior findings showing that such complex amine functionalities promote stronger interactions with more hydrophobic, monovalent anions such as ReO_4_^−^, while exhibiting lower affinity for multivalent and more hydrophilic anions such as sulfate, molybdate, or arsenate species [40]. In this context, resins bearing tertiary or heterocyclic amine groups have been observed to discriminate in favor of ReO_4_^−^ because of both hydrophobic interactions and steric effects, which can modulate ion-exchange equilibria [4,40,41].

Hence, the objective of this study was to synthesize and characterize a series of novel anion-exchange resins modified with a range of WB amine groups of the aliphatic, heterocyclic, and aromatic varieties. The thus-characterized resins were then evaluated in terms of the selective adsorption of ReO_4_^−^ ions from simulated Cu-Mo ore processing solutions. We hypothesized that the thus-developed anion-exchange resins could significantly improve both the adsorption capacity and selectivity of Re(VII) ion recovery, even in the presence of competing ions such as Cu, Mo, and V.

## 2. Results and Discussion

### 2.1. Synthesis of Anion-Exchange Resins

**Figure 1:**
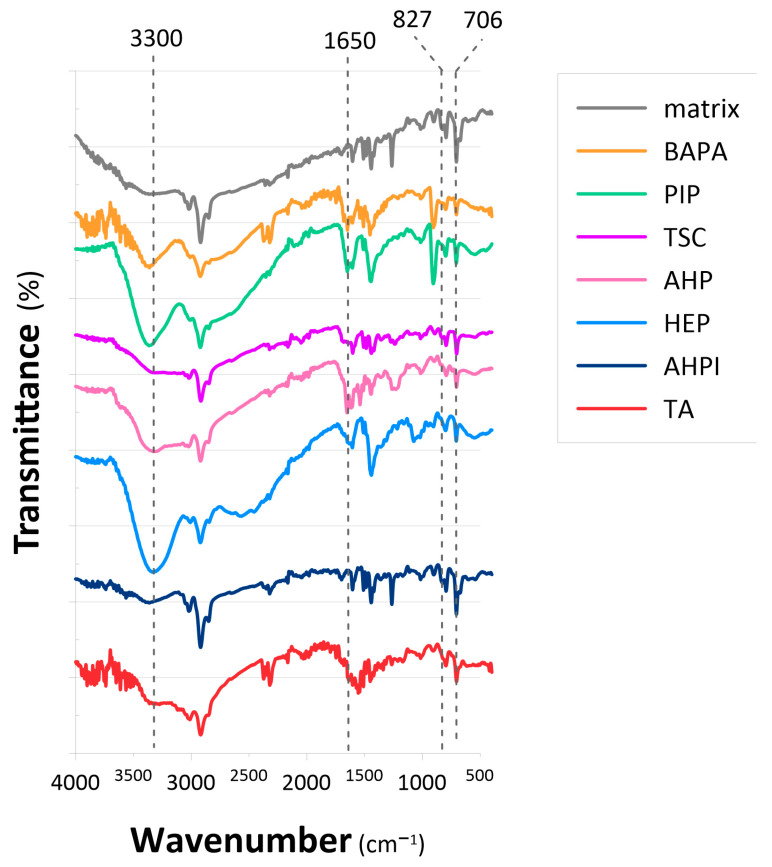
ATR-FTIR spectra of the polymeric matrix and anion-exchange resins derived therefrom.

Because the aforementioned hypothesis, serving as the motivation for this study, is based on the premise that the adsorption of Re can be enhanced by weakly basic amines characterized by a complex structure [4,40], a series of new anion-exchange resins was synthesized. These resins were obtained by applying various aliphatic, heterocyclic, and aromatic amines. To verify whether the introduction of amino functionalities into the VBC-co-DVB copolymer was successful, both the polymeric matrix and the synthesized anion-exchange resins were analyzed using ATR-FTIR. Figure 1 displays the ATR-FTIR spectra of all the synthesized anion-exchange resins.

The spectrum of the VBC-co-DVB matrix reveals the presence of bands located at 827 and 706 cm^−1^ that are likely due to C–Cl vibrations in the –CH_2_Cl groups derived from the VBC monomer [42]. After the modification of the matrix, the first band faded, while the second one decreased in intensity (, and a series of new bands appeared in the N–H stretching region (3377–3300 cm^−1^) and in the N–H bend region (~1650 cm^−1^). The latter bands were found in all the anion-exchange resins, although with slightly varied wavenumbers. Furthermore, the TSC and TA resins also revealed the characteristic bands that could be associated with the S–H and C–S stretching vibrations that were found at ~700 cm^−1^ [42], respectively (. However, it must be noted that these could overlap with the unreacted –CH_2_Cl groups. All these observations indicated that the amino functionalities were indeed introduced into the polymeric matrix. The ATR-FTIR spectra, further supported by the test revealing or excluding the presence of –NH_2_ groups (see Figure S1 for details), allowed us to confirm the predicted structures of the anion-exchange resins.

To enhance the interpretation of FTIR spectra, Principal Component Analysis (PCA) was performed on the spectral data collected for the synthesized resins and the unmodified copolymer matrix. The data matrix consisted of transmittance values over the full spectral range (4000–400 cm^−1^) for each resin. The first two principal components (PC1 and PC2) explained a significant portion of the total variance, collectively accounting for approximately 18–59% of the spectral variability. The score plot (Figure S2) revealed clear separation between the unmodified matrix and the functionalized resins, indicating distinct chemical functionalities introduced by amination. Notably, the samples modified with heterocyclic, aromatic amines, and aliphatic amines were well separated, highlighting differences in their vibrational features. This result supports the conclusion that the chemical structure of the amines significantly influences the resulting polymer matrix and validates the successful incorporation of diverse functional groups.

The elemental analysis results given in Table 1 reveal that the anion-exchange resins contain N, S, and O at the concentrations expected to be found (Figure 1 and Table 1), constituting yet another piece of evidence of successful modification. Interestingly, however, the Hecker’s anion-exchange capacity determined (Z_H_, Table 1) showed significant differences. Because Hecker’s method involves the determination of ionic Cl discharged from functionalities, the Z_H_ value directly accounts for the ability of amines to dissociate. The Z_H_ was determined to range from 0.04 to 0.26 mmol g^−1^ and was the highest in the case of the BAPA, PIP, and HEP resins. For the remaining resins (TSC, AHP, and TA), the Z_H_ was about five times smaller than expected in reference to the N concentration determined in these resins, suggesting that the TSC, AHP, and TA functionalities, although successfully introduced, were not able to dissociate or dissociated poorly. As such, it was presumed that these resins, although characterized by the desired structure, would exhibit negligible ion-exchange capacity; hence, they were excluded from further studies.

**Figure 2:**
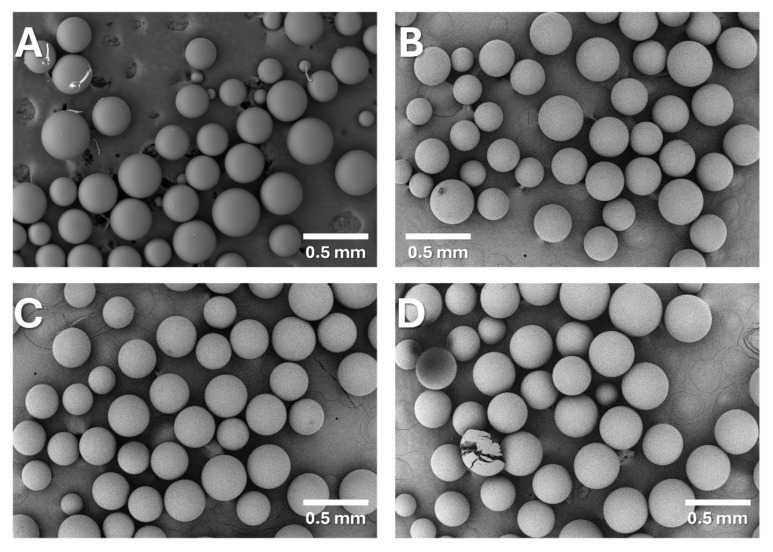
SEM images of (**A**) the VBC-co-DVB copolymer and (**B**) the BAPA, (**C**) PIP, and (**D**) HEP anion-exchange resins.

As such, only the BAPA, PIP, and HEP resins possessing a certain anion-exchange capacity were selected for further characterization. The morphologies of the BAPA, PIP, and HEP resins were assessed using scanning electron microscopy (SEM). Figure 2 displays the corresponding SEM images captured for the polymeric matrix and the Re-loaded BAPA, PIP, and HEP anion-exchange resins. Furthermore, Table S1 displays the elemental compositions of the surfaces of these resins, as measured via EDX.

Based on the SEM/EDX analysis, it appeared that the suspension copolymer, as well as the anion-exchange resins based on it, were spherical and had a uniform morphology characterized by beads of a diameter ranging from 0.1 to 0.4 mm, and no porosity was observed (Figure 2). Hence, the predicted expanded-gel structure of the polymers was indeed created. The data provided in Table S1 for the resins loaded with Re confirm their ability to adsorb this metal from the solution because they indeed contained N-based species on their surfaces. Simultaneously, the initially observed Cl concentration for the VBC-co-DVB copolymer (23.56%) was found to decrease to 8.62–13.19% (Table S1), a result consistent with the changes in the ATR-FTIR spectra observed for the –CH_2_Cl groups after their modification. Simultaneously, significant concentrations of Re were noted in the case of the BAPA and PIP resins, suggesting they were able to adsorb this metal. Although no Re was detected on the surface of the HEP resin, based on previous results, we expected this anion-exchange resin to operate efficiently in the Re adsorption processes. The absence of Re in the EDX spectra could be due to an excessive coverage of the HEP resin sample by C upon its preparation (Table S1). Hence, we decided to use the HEP resin together with the BAPA and PIP resins for studies on the adsorption process.

At this point, it should also be noted that that the selection of toluene as a reaction environment during the synthesis of the VBC-co-DVB copolymer induced the formation of an expanded gel structure of the synthesized material, whose formation was verified by carrying out a control experiment involving low-temperature adsorption and desorption that confirmed the complete lack of porosity in the investigated sample.

### 2.2. Resin Dose and Optimal Adsorption pH

**Figure 3:**
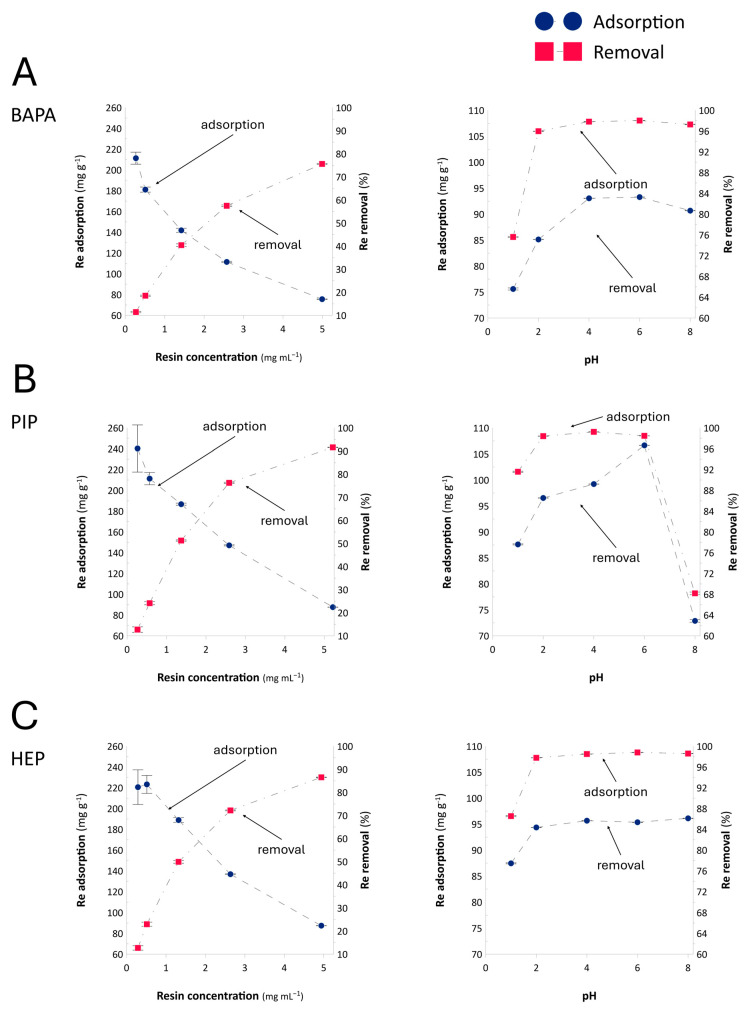
The adsorption rate (mg g^−1^) and removal efficiency (%) of the Re(VII) ions as a function of resin concentration and solution pH determined for the process carried out over the (**A**) BAPA, (**B**) PIP, and (**C**) HEP anion-exchange resins. The initial Re(VII) concentration was 500 mg Re L^−1^.

Based on the results obtained, we expected the novel BAPA, PIP, and HEP anion-exchange resins to be able to reveal the anion-exchange capacity toward ReO_4_^−^ ions. However, before studying the adsorption equilibrium, we selected the resin dose and the optimal adsorption pH. Accordingly, the anion-exchange resins were placed in contact with Re(VII) solutions under varying adsorbent masses and solution pH values. Figure 3 displays plots representing Re adsorption (mg g^−1^) and the removal of this metal (%) as a function of the anion-exchange resin concentration and solution pH, respectively.

Based on the results (Figure 3), we concluded that the optimal ion-exchange resin dosage for all the tested polymers was 5 mg mL^−1^ (100 mg per 20 mL of solution), based on the fact that the sorption curves reached a plateau at this dosage. The PIP resin demonstrated the highest ReO_4_^−^ removal efficiency (92%), followed closely by the HEP resin (87%), while the BAPA resin exhibited the lowest removal efficiency (76%) (Figure 3). These values correspond to the adsorption of 240, 220, and 211 mg of Re g^−1^, respectively. Based on these observations, we expected the PIP resin to be the most effective for Re adsorption.

When considering the adsorption process as a function of solution pH (Figure 3), the tested anion-exchange resins exhibited adsorption capacities in the range of 75.6 to 106.6 mg g^−1^ across the pH range of 1 to 8. The BAPA resin showed the highest sorption at pH 6 and almost the same value at higher pH levels; at pH 1, this sorption figure was about 19% lower than the highest value. These results suggest that the optimal application for the BAPA resin should fall within the pH range of 2 to 8. Similarly, the PIP resin reached maximum sorption at pH 6 (98.5%), though it showed a minimum at pH 8 (68.4%) and a lower capacity at pH 1 (82.2%). As such, this resin is suitable for ion exchange within a narrower pH range of 2 to 6. In contrast, the HEP resin achieved maximum sorption at pH levels in the range of 2–8 and the lowest sorption at pH 1 (91%), with an Re removal capacity of 99.3% at pH 6. Although this resin exhibited about 8% lower adsorption at pH 1, it appears to be the most versatile, as it exhibited similar effectiveness across the widest pH range, that is, 2 to 8.

The observed decrease in adsorption capacity at pH 1 for all the tested resins may be attributed to osmotic effects rather than changes in the ion-exchange mechanism. At highly acidic pH values, osmotic shock can occur because of the large concentration gradient between the resin phase and the external solution [43], leading to reduced resin swelling and, consequently, limited accessibility of functional groups for ReO_4_^−^ ions. In fact, the solvent regain estimated for the anion-exchange resin appeared to be 6–10% smaller (0.17, 0.09, and 0.07 g HCl g^−1^ for BAPA, PIP, and HEP, respectively) than the water regain (Table 1). In contrast, the difference in adsorption behavior among the resins at pH 8 appears to be related to the chemical nature of their functional groups. While the BAPA and PIP resins rely on primary and secondary amines (Figure 1), which gradually lose their protonation and ion-exchange capacity as the pH increases [43], the HEP resin contains hydroxyethyl-substituted amines (Figure 1). This structural feature likely stabilizes the resin’s swelling and hydration properties, allowing it to maintain efficient ReO_4_^−^ adsorption, even at higher pH values.

Considering these data and the practical applications of the resins in ion-exchange columns for industrial use, the resins are potentially suitable for technologies related to the processing of Cu-Mo leachates. Because these technologies, depending on the strategy employed, involve leaching with H_2_SO_4_ [4,35], the pH of these systems can be estimated to fall within the range of ~0.5–2. In this study, the results of the test carried out at pH 1 (Figure 3) suggest the synthesized resins could indeed operate under real-life conditions. However, because the maximum sorption capacities were observed at higher pH values, research focused on the adsorption equilibria was conducted to determine the optimal conditions. Then, to enable us to draw conclusions on potential industrial applicability, we carried out all subsequent research at pH 1.

### 2.3. Adsorption Equilibrium

**Figure 4:**
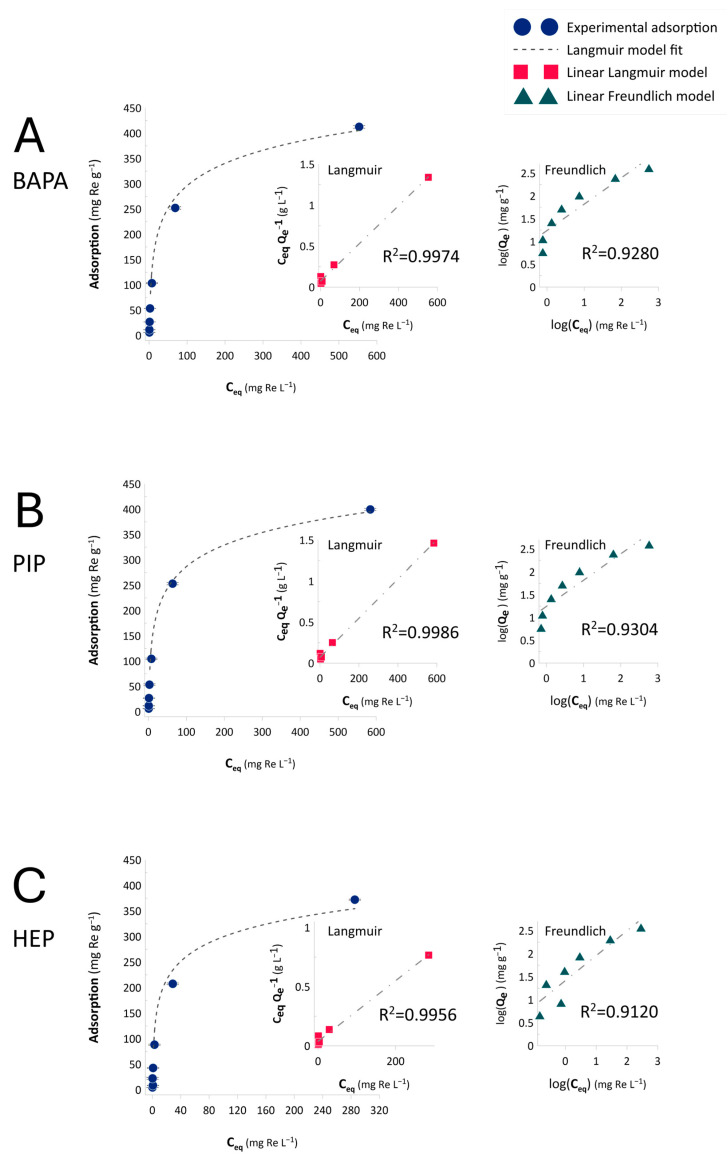
The Re(VII) ion adsorption (mg g^−1^) isotherms and the Langmuir and Freundlich fits for the process carried out on the anion-exchange resins (**A**) BAPA, (**B**) PIP, and (**C**) HEP. The resin dose was 5 mg mL^−1^ at pH 6.

Figure 4 displays the Re(VII) ion adsorption isotherms acquired for the tests carried out on the BAPA, PIP, and HEP anion-exchange resins. The experimental data were fitted with the Langmuir (Equation (3)) and Freundlich (Equation (4)) models, whose linear regressions are also displayed in Figure 4.

Based on the results, Re(VII) ion adsorption obeyed the Langmuir model, indicating that the process revealed a chemical character and the anticipated anion exchange of ReO_4_^−^ ions. As displayed in Table 2, the maximum adsorption capacities achieved by the tested anion-exchange resins ranged from 390.5 to 435.4 mg Re g^−1^. These values were consistent with the Z_H_ values (Table 1) and could be ordered as follows: BAPA > PIP > HEP. All the resins exhibited the similar binding-constant (K_L_, Table 2) values, which, combined with the separation factors (R_L_, Table 2), falling within a range of 0–1, allowed us to describe the extent of Re(VII) ion adsorption as “favorable” [44,45].

At this point, it should also be noted that, although all the synthesized resins were derived from the same VBC-co-DVB matrix, differences in functionalization efficiency were observed, as reflected by the elemental nitrogen content (Table 1). In this context, no direct correlation between the total nitrogen content and Re(VII) adsorption capacity was identified, indicating that not all the introduced amine functionalities were available to perform anion-exchange. However, a link was found between the Hecker’s anion-exchange capacity (Z_H_, Table 1) and the maximum Re(VII) adsorption capacities determined from the Langmuir isotherms. The resins with higher Z_H_ values, reflecting the number of dissociable amine groups available for anion exchange, exhibited correspondingly higher adsorption capacities. This finding suggests that the functionalization efficiency relevant to Re(VII) recovery depends not only on the amount of nitrogen introduced into the polymer matrix but also on the chemical nature and accessibility of functional groups capable of participating in ion-exchange processes.

**Figure 5:**
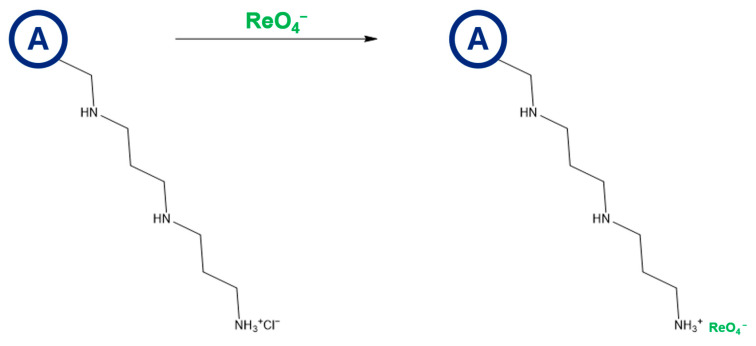
The anticipated anion-exchange reaction occurring over the BAPA anion-exchange resin.

Based on the results described above, the extent of the amino groups’ protonation (Table 1, N% vs. Z_H_), and the fact that HCl was used to adjust the Re(VII) solution’s pH, we were able to propose the anion-exchange reaction occurring on the surfaces of the resins. Figure 5 displays the predicted anion exchange between the BAPA resin and the ReO_4_^−^ oxyanions.

### 2.4. Adsorption Selectivity and Re(VII) Elution

Because Re exists only in trace levels in Mo-Cu ores [3,5,15,46] and is accompanied by Mo(VI), V(V), and Cu(II) species, we aimed to develop anion-exchange resins that could be well suited for such Re sources. Based on the results of the equilibrium studies, the synthesized polymers BAPA, PIP, and HEP revealed a capacity for anion exchange toward the ReO_4_^−^ ions, with outstanding amounts of Re adsorbed (up to 435 mg g^−1^). To verify the suitability of the resins for simulated industrial conditions, the BAPA, PIP, and HEP resins were placed in contact with an acidic 1.4 mmol L^−1^ Re(VII) solution (0.1 mol L^−1^ HCl, pH 1), to which an equivalent amount of Mo(VI) (1.4 mmol L^−1^) and excess amounts of V(V) and Cu(II) (4 mmol L^−1^ each) were introduced.

**Figure 6:**
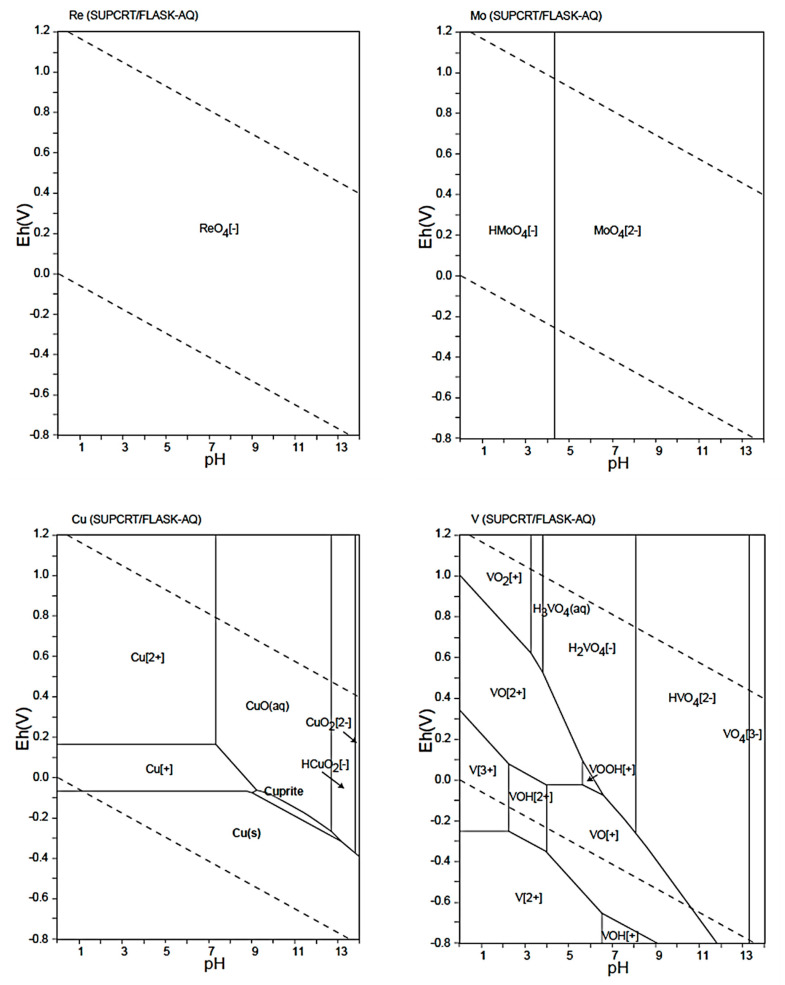
Eh-pH diagrams of Re, Mo, V, and Cu species. These diagrams were reproduced based on ref. [47] via the Geological Survey of Japan Open File Report No. 419.

To support the following results and discussion, the speciation of the target metals must be considered. Figure 6 displays Eh-pH diagrams of Re, Mo, V, and Cu species [47].

Based on the data displayed in Figure 6, the prepared multicomponent solution in 0.1 mol L^−1^ (pH 1) should contain Cu^2+^ and a variety of V cationic species, including V^2+^, V^3+^, VO_2_^+^, and VO^2+^ (Figure 6) [47]. Simultaneously, Re has revealed a tendency to be stable over a wide range of pH ReO_4_^−^ forms, while Mo can exist in the form of HMoO_4_^−^ or, according to some databases, MoO_4_^2−^ [47]. In this context, because Cu and V were expected not to compete with Re to access the anion-exchange centers, they were used as “ballast” metals. Hence, to simulate real-life conditions, the concentration thereof was set to 4.0 mmol L^−1^. Simultaneously, the concentration of Re was set at a lower level, 1.4 mmol L^−1^, and the concentration of Mo was set at the same level to highlight potential competitivity for the resins’ functionalities.

**Figure 7:**
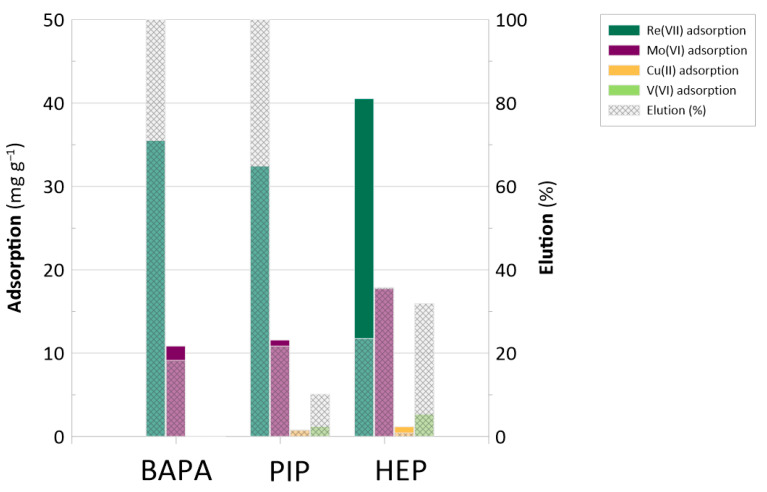
The adsorption rates (mg g^−1^) and elution (%) of Re, Mo, Cu, and V achieved for the BAPA, PIP, and HEP functionalities.

Considering the results regarding the adsorption rate (mg g^−1^) and the residual concentrations (mg L^−1^) of Re(VII), Mo(VI), Cu(II), and V ions, we calculated the partition coefficients (K_D_ (Equation (9))) and selectivity indices (S_i_ (Equation (10))). Figure 7 displays the adsorption rates (mg g^−1^) and elution percentages for Re, Mo, Cu, and V.

As shown in Figure 7, the highest Re adsorption rate values were recorded for the multicomponent solution. The corresponding values ranged from 32 to 41 mg Re g^−1^. These values were consistent with the maximum adsorption capacity of the resins recorded for pH 1 (Figure 3); however, the adsorption of Mo(VI) also occurred. The adsorption rates ranged from 10–17 mg Mo g^−1^, 2–3 times lower than the adsorption rate of Re. Figure 7 also evidences that the BAPA, PIP, and HEP anion-exchange resins preferred to uptake Re over other metallic species present in the solution. In the case of Cu and V, the adsorption rate values were negligible; as expected, at pH 1, these metals form cations instead of anionic complexes (Figure 6) [47].

The opposite situation was expected in the case of Mo(VI) adsorption, as these species are expected to be anionic in nature (HMoO_4_^−^ and MoO_4_^2−^ [47]). However, based on the selectivity indices displayed in Table 3, the uptake of Re was about 2–3 times greater than that of Mo. This result suggests that the resins indeed exhibited selectivity to Re over Mo species. This dependence could be associated with several factors. (1) The first concerns species valency: the bivalent MoO_4_^2−^ ions [47] might have been adsorbed to a degree at least 2 times lower than that of the monovalent ReO_4_^−^ ions. (2) The second concerns hydration energies: MoO_4_^2−^ exhibits a higher hydration energy than ReO_4_^−^ [48], indicating a stronger interaction with water molecules. This makes Mo species “more hydrophilic”. Conversely, ReO_4_^−^ ions have a lower hydration energy [48], making them relatively “more hydrophobic”. This difference influences their behavior in aqueous systems, particularly in separation processes at the solid–liquid interface. That is, ReO_4_^−^ might have just revealed greater affinity toward the hydrophobic matrix of the functionalized resins. (3) The third factor relates to the structures of the functionalities: MoO_4_^2−^, owing to its “more hydrophilic” nature, tends to become more hydrated [48,49] and thus could suffer from steric hinderance in the amino-functionalized polymeric matrix, making the above-mentioned limited migration of Mo species within the resin even more difficult. Considering the aforementioned information, it seems that the hypothesis of this study has indeed been confirmed. The “complex” amino functionalities, possessing developed aliphatic chains and substituents, as well as heterocyclic and aromatic rings, might have been a direct cause of the resins’ preference for ReO_4_^−^ over Mo species.

Despite the above-mentioned considerations regarding the selectivity of the resins, the developed BAPA and PIP resins also appeared to effectively recover Re, as the applied strategy resulted in the complete (100%) elution of Re from the surfaces of these resins (Figure 7). However, it must be noted that only just over 20% of the Re loaded onto the HEP resin was stripped from its surface. Based on previous investigations involving anion-exchange resins functionalized with electron-donating groups, a possible explanation of this phenomenon could be that the HEP resin is able to perform in situ reduction of Re(VII) to lower oxidation states [50]. Figure S3 displays a Re 4f spectrum recorded in our previous studies for the same sample. Based on this, the HEP resin can reduce Re^+7^ to Re^+4^ and Re^+6^ species. In previous work, we carried out deliberate Re(VII) reduction under conditions similar to those applied in this study between adsorption–desorption cycles. The HEP sample seems to promote the aforementioned reduction because the HEP functionality contains hydroxyl (–OH) groups capable of acting as mild reducing agents in aqueous environments. It has been shown to promote the reduction of metal chlorocomplexes and oxoanions, including AuCl_4_^−^ and ReO_4_^−^, under similar conditions [50,51,52]. Accordingly, we cannot exclude the possibility that a similar reduction pathway occurred here, limiting the efficiency of Re(VII) desorption from the HEP resin. While direct experimental confirmation of this mechanism is outside the current scope, its existence is supported by extensive prior work on structurally related systems and functional groups. At this point, it is also worth noting that undesired (in the present scenario) reduction may be controlled by decreasing the pH of the solution in which adsorption takes place [53].

### 2.5. Industrial Implications

Notably, the synthesized anion-exchange resins are based on the VBC-co-DVB copolymer, which exhibits structural characteristics shared by commercial styrene-based ion exchangers. Such materials are widely known for their robustness and have demonstrated high mechanical and thermal stability in industrial applications, even under continuous-flow conditions and in the presence of acidic media. Their performance in demanding operational environments has been extensively validated, making them a reliable platform for real-life applications in ion-exchange technologies [54]. Furthermore, it should also be noted that the synthesized resins are based on a cross-linked VBC-co-DVB copolymer matrix that is the same as a number of commercial products or very similar to the majority of S-co-DVB matrices that are already used at a large scale in the manufacture of ion-exchange resin. Hence, they share primary raw materials that are similar to or the same as those of commercial resins. In this context, while exact synthesis costs can vary based on scale and regional factors, given the similarity of the base materials and synthesis processes, the cost of the synthesized resins is anticipated to be within a range comparable to that of the commercial products, especially when produced at scale.

Besides these considerations, there are several practical aspects that could be addressed to assess the potential industrial relevance of the proposed anion-exchange resins. These aspects include the assessment of adsorption kinetic behavior, the resins’ stability, and their suitability for continuous-flow-mode adsorption. Based on the results above, the BAPA resin was selected as a representative for the subsequent research.

**Figure 8:**
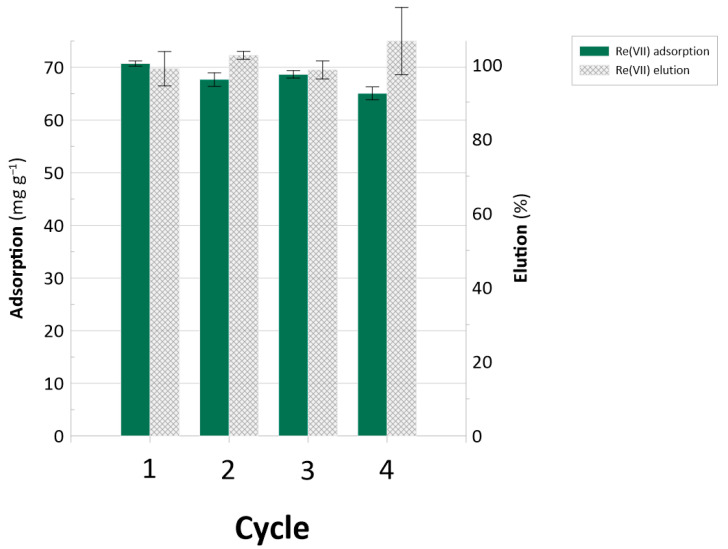
Cyclic Re adsorption and elution for the BAPA sample.

To assess the resin’s stability, a test of cyclic Re adsorption and elution was performed. Figure 8 displays adsorption and Re stripping values for over four adsorption–elution cycles.

Based on the results, the BAPA resin was stable over the tested adsorption–desorption cycles. The adsorption capacities ranged from 65 to 70 mg Re g^−1^. At this point, it is worth noting that the elution tests carried out using the multicomponent solutions (Figure 7) were performed using 1 mol L^−1^ of NaOH as a stripping agent in order to ensure that all the metal species would be released from the surface of the resin under the test conditions. Here, even though a much milder stripping agent was applied (0.1 mol L^−1^ NaOH), each cycle resulted in 100% Re elution (Figure 8). All of these observations further indicate the practical potential of the BAPA resin.

**Figure 9:**
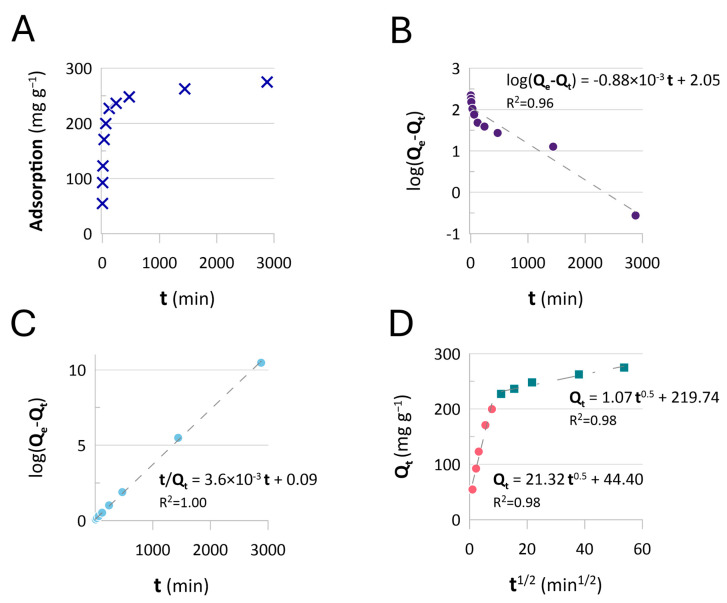
Kinetics of Re(VII) adsorption over BAPA resin: (**A**) Re(VII) adsorption as a function of time and plots of the kinetics models applied: (**B**) PFO, (**C**) PSO, and (**D**) intraparticle diffusion kinetics, respectively. The resin dose was 5 mg mL^−1^, at pH 1; the initial concentration was as follows: C_0_ = 1500 mg Re L^−1^.

The kinetic behavior of the BAPA samples was evaluated in terms of the pseudo-first-order and pseudo-second-order models (PFO and PSO) and the intraparticle diffusion model. These are displayed as model fits in Figure 9.

Based on the data obtained, the adsorption equilibrium was reached 10 min after the beginning of the process (Figure 9A). These data were then applied to the pseudo-first-order (PFO, Figure 9B) and pseudo-second-order (PSO, Figure 9C) kinetic models. The PSO model yielded correlation coefficient (R^2^) values of 1 (Figure 9C), suggesting the adsorption indeed obeyed the anion-exchange mechanism [55,56], as predicted in the tests on adsorption equilibria. Furthermore, under the applied conditions, the expected maximum adsorption capacity derived from the PSO model was 275 mg Re g^−1^. Notably, this value is 38% lower than the maximum adsorption predicted by the Langmuir isotherm model (435 mg Re g^−1^, Table 3). It must be remembered, however, that the equilibrium studies were carried out at pH 6, determined to be optimal for the adsorption process over BAPA samples. Here, the test carried out at pH 1 could be considered to reflect more industrially oriented acidic conditions, under which the value of adsorption is still more than satisfactory. This, however, will be shown through other Re-recovery-oriented studies later in this section.

The kinetic data were then applied to the intra-particle pore diffusion (IPD) model. Based on the fits displayed in Figure 9D, the Re(VII) adsorption mechanism involved two steps. The first, which is steeper, can be associated with film diffusion; the second step is probably associated with intraparticle diffusion, i.e., resin saturation [57]. In this scenario, the intercepts (Figure 9D) correspond to layer thickness, which played a role in Re(VII) bonding. This result, linked with the rate value determined by the slope of the first stage of adsorption (Figure 9D) and a slight curvature observed between 7 and 15 min^−0.5^ (Figure 9D), suggests that the adsorption rate is influenced by external mass transfer, allowing us to conclude that the process occurring at the solid–liquid interface is quick [57]. This must be due to the expanded-gel structure of the matrix applied for the synthesis of the anion-exchange resins. Lack of porosity apparently marginalized the diffusion limitations; as such, we expected the BAPA resin to be a good fit for continuous-flow-mode systems.

**Figure 10:**
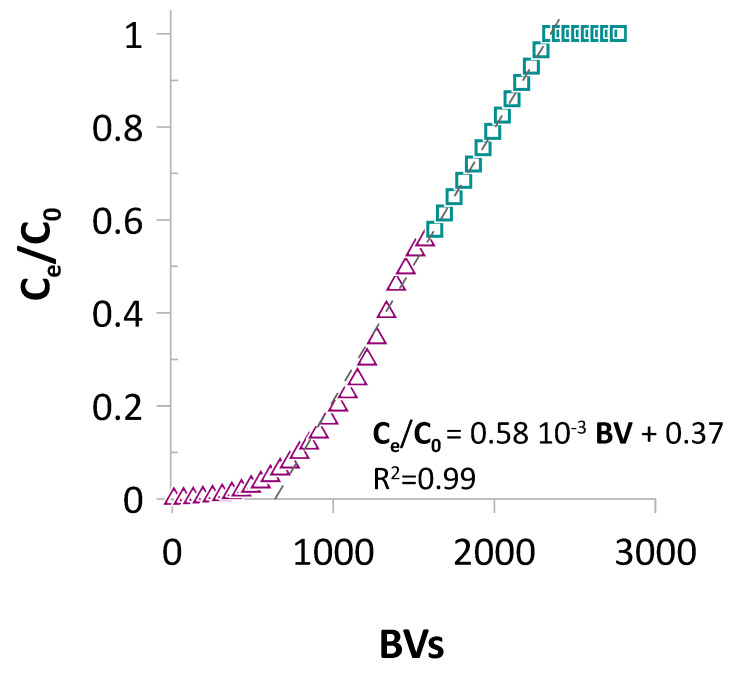
BAPA-loaded column breakthrough recorded during ReO_4_^−^ continuous-flow-mode adsorption. *C*_0_ = 100 mg Re L^−1^; bed volume (*BV*): 1.5 mL; flow rate: 60 BVs h^−1^.

In this context, the BAPA resin was applied in this system, and the flow-mode process was carried out. Figure 10 displays the ReO_4_^−^ adsorption curve recorded during the test.

Based on the data collected, the BAPA resin can process high volumes of Re(VII) solution. The test was carried out at up to 1600 BVs (equal to 2400 mL of the feeding solution) at 57% column breakthrough, while 1% and 5% breakthrough points were observed at 340 and 640 BVs, respectively (Figure 10). These results can be considered as outstanding, especially when considering the potential scale of a real process. Notably, a quick look at the guides provided by commercial resin producers suggests flow rates ranging from 10 to 50 BVs h^−1^ depending on process conditions [58]. In the present scenario, the applied flow rate through the column was 60 BVs h^−1^.

The test was conducted at up to 1600 BVs, at which point we reached capacity of our system. Because of this limitation, a linear approximation was applied to the breakthrough data, allowing us to draw an anticipated sigmoidal shape of the curve up to the point of complete exhaustion of the resin. Based on these data, we predicted that the BAPA resin would completely lose its capacity at 2350 BVs (Figure 10).

It is worth highlighting that the novel anion-exchange resins developed in this study demonstrate superior performance relative to previously reported materials. Specifically, as displayed in Table 4, these resins exhibit significantly higher adsorption capacities (up to 435 mg Re g^−1^,) than commercial resins or those reported in the literature, such as Purolite A170 (~100 mg Re g^−1^) [4], modified D201 (92.16 mg Re g^−1^) [35], or even the high-performing D990 resin (162 mg Re g^−1^) [59]. The selectivity indices of 2.9 and 1.9 indicate a notable preference for rhenium over competing molybdenum ions, a property that is crucial for processing Re-bearing leachates from Mo ores. Although these values are much smaller than the ones achieved in our previous study [41] for the 1-(3-aminopropyl)imidazole-modified resin, this phenomenon underlines the importance of further developing and optimizing such materials. Additionally, the BAPA resin presented in this study exhibits excellent regeneration efficiency, with nearly 100% Re elution maintained over at least five sorption/desorption cycles, outperforming other systems, such as D201 (~95%) and D990 (97.3%). These advantages, coupled with stable operation in continuous flow mode, suggest that the newly developed resins offer a significant advancement in rhenium separation technology, particularly for sustainable and scalable applications.

It must also be noted that while the synthesized anion-exchange resins demonstrated stable performance within the tested pH 1, certain limitations related to long-term chemical and mechanical stability should be considered. Prolonged exposure to strongly alkaline or oxidative environments may lead to degradation of the styrenic matrix or functional groups. Additionally, mechanical stability under continuous-flow conditions beyond the laboratory scale, as well as resistance to fouling caused by organic matter present in real industrial solutions, remains to be evaluated in future studies.

## 3. Experimental Section

### 3.1. Materials

The vinylbenzyl chloride-co-divinylbenzene copolymer (VBC-co-DVB) was synthesized using analytical-grade (VBC, meta and para isomers) and technical-grade (DVB, 80%) monomers. Initially, the monomers were purified from inhibitors via vacuum distillation. The amines (analytical grade) applied for the modification of the VBC-co-DVB copolymer were used as received. Analytical-grade ammonium perrhenate(VII) (NH_4_ReO_4_), ammonium metavanadate(V) (NH_4_VO_3_), ammonium molybdate(VI) ((NH_4_)_6_Mo_7_O_24_⸱4H_2_O), and copper(II) sulphate (CuSO_4_⸱5H_2_O) were used to prepare Re(VII), V(V), Mo(VI), and Cu(II) stock solutions used for the adsorption studies. All these reagents were acquired from Sigma Aldrich Chemical Co. (Merck branch Poland, Warsaw, Poland). All other reagents and solvents (analytical-grade or better) applied in this study were purchased from Avantor Performance Materials Ltd. (Gliwice, Poland) and used as received. Reverse-osmosis water was used throughout all the experiments.

### 3.2. Synthesis of Anion-Exchange Resins

**Figure 11:**
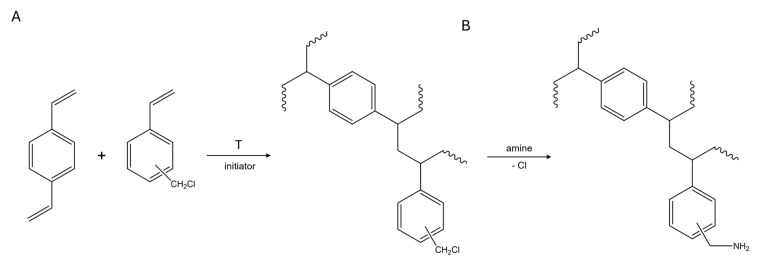
Simplified route of anion-exchange resin synthesis: (**A**) polymerization of VBC and DVB, and (**B**) amination of VBC-co-DVB copolymer.

Figure 11 displays a simplified scheme of the procedure applied for anion-exchange resin synthesis performed according to the methodology described in this section.

In the first stage, the VBC-co-DVB polymeric matrix was synthesized by applying the suspension polymerization technique. The organic phase consisted of toluene; a free-radical initiator, namely, benzoyl peroxide (BPO); and the mixture of VBC and DVB (2 mol% with respect to VBC). The thus-prepared organic phase was suspended in the water phase, consisting of poly(vinyl)alcohol and CaCl_2_. First, the water-phase material was placed in a glass reactor and heated to 50 °C. Constant stirring (250 RPM) was applied. Then, the organic phase was introduced into the reactor, and the whole mixture was purged with N_2_. Afterward, the stirring rate was increased to 300 RPM, and the temperature was gradually increased to 90 °C. This suspension polymerization was carried out for 25 h. Afterward, the resultant VBC-co-DVB suspension copolymer (Figure 11A) was extracted into toluene, dried, and used for the modification procedure.

**Figure 12:**
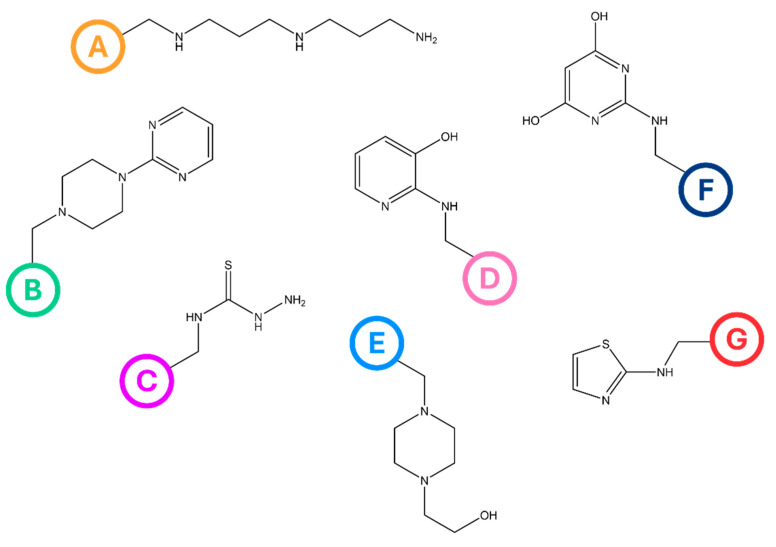
The predicted structures of the (**A**) BAPA, (**B**) PIP, (**C**) TSC, (**D**) AHP, (**E**) HEP, (**F**) AHPI, and (**G**) TA anion-exchange resins.

The applied suspension polymerization technique allowed us to obtain polymer beads that exhibited a uniform spherical morphology, with diameters ranging from 0.1 to 0.4 mm. Toluene was applied as the organic-phase component, serving as a “good” solvent with respect to VBC and DVB, and it enabled the formation of an expanded-gel structure of the polymeric matrix. The resultant polymer contained chloromethyl groups introduced via VBC that further served as anchoring points for functionalization. As such, the modification of the VBC-co-DVB copolymer was carried out in the following way (Figure 11B). The dry polymeric matrix was swollen in dimethyl formamide, and then amines, i.e., bis(3-aminopropyl)amine (BAPA), 1-(2-pyrimidinyl)piperazine (PIP), thiosemicarbazide (TSC), 2-amino-3-hydroxypyridine (AHP), 1-(2-hydroxyethyl)piperazine (HEP), 4-amino-2,6-dihydroxypyrimidine (AHPI), and 2-thiazolamine (TA), were introduced into the swollen polymer in an amount that ensured there was a 5:1 amine-to-chlorine ratio in the polymeric matrix. The thus-prepared mixtures were then left for 14 days under ambient conditions on an orbital shaker. In this way, the simplified synthetic route depicted in Figure 11 allowed us to obtain a series of anion-exchange resins, coded BAPA, PIP, TSC, AHP, HEP, AHPI, and TA, whose predicted structures are displayed in Figure 12.

Afterward, the resultant resins were filtrated, washed with water, and then successively washed with 1 mol L^−1^ HCl, 1 mol L^−1^ NaOH, and H_2_O. Finally, after the last wash with 1 mol L^−1^ HCl, the polymers were washed with 0.001 mol L^−1^ HCl and used for further analysis.

### 3.3. Characterization Methods and Instrumentation

The morphologies of the anion-exchange resins were assessed using scanning electron microscopy (SEM). The SEM images were taken using a Hitachi S-3400N (Hitachi Ltd., Tokio, Japan). An elemental analysis (EA), including C, H, N, S, and O, was performed with the aid of a Vario EuroVector 3000 analyzer (Elementar Analysensysteme GmbH, Langenselbold, Germany). The structures of the functionalities introduced into the polymeric matrix were confirmed using Fourier-transform infrared spectroscopy (FT-IR). The FT-IR spectra were recorded using a Jasco ATR-FTIR spectrometer, model FT-IR 4700 (Medson, Paczkowo, Poland), operating in the range of 4000–400 cm^−1^ and taking 64 scans per measurement, with a resolution of 4 cm^−1^.

To deepen the interpretation of the FTIR spectral data, multivariate statistical analysis was applied using Principal Component Analysis (PCA). The transmittance values collected from the spectra in the range of 4000–400 cm^−1^ were arranged into a matrix, where each row represented a different polymer sample, and each column corresponded to a specific wavenumber. Prior to the PCA, the data were mean-centered, and no additional scaling was applied. The PCA was conducted using Python 3.11.8 (Python Software Foundation, Wilmington, DE, USA) libraries (NumPy 1.24.0, Pandas 1.5.3, and scikit-learn 1.1.3), facilitated through the use of the ChatGPT 4o model (GPT-4 Turbo, August 2025 version, OpenAI, San Francisco, CA, USA) to assist with data processing, Python code generation, and visualization.

The water regain (g g^−1^) of the anion-exchange resins was determined by calculating the mass balance between centrifuged wet and dried samples of these resins. The anion-exchange capacity of the resins (Z_H_) was determined using Hecker’s method [62,63,64], which allows one to estimate the number of N atoms in the amino functionalities to be dissociated. We used these tests to pre-screen the prepared anion-exchange resins and determine which ones were appropriate for use in the Re adsorption studies. The tests were repeated twice for each polymer sample. The concentrations of Re, Mo, V, and Cu were determined using inductively coupled plasma optical emission spectrometry (ICP-OES) via an Agilent 5110. The adsorption rates of these metals (mg g^−1^) were calculated from the mass balance based on measurements repeated three times. The presence of –NH_2_ groups in the anion-exchange resins was confirmed by carrying out a colorimetric test in the presence of ninhydrin [64].

### 3.4. Adsorption Equilibrium and Kinetics

All the tests on the Re(VII) adsorption were carried out at ambient temperature (20 °C). First, we determined the adsorption of Re(VII) ions as a function of anion-exchange resin concentration by taking resin doses in the range of 5–100 mg and placing them in 20 mL of 500 mg Re L^−1^ in a 0.1 mol L^−1^ HCl solution (pH 1). The thus-prepared samples of the anion-exchange resins were equilibrated for 48 h on an orbital shaker and separated via filtration, while the Re concentration in the resulting filtrates was determined using ICP-OES.

Secondly, the pH of the solutions used for Re adsorption was adjusted. To achieve this, the pH of 500 mg of Re L^−1^ in a H_2_O solution was adjusted using HCl and NaOH. Then, 100 mg (dry mass) of a given anion-exchange resin was introduced into the Re(VII) solutions, with the pH ranging from 2 to 8. Then, the samples of the anion-exchange resin were equilibrated and separated via filtration, while the resulting filtrates were subjected to ICP-OES analysis to determine the Re concentration.

The thus-obtained results were re-calculated to determine Re(VII) adsorption (mg g^−1^, Equation (1)) and removal (%, Equation (2)):

(1) $$S(mg\;g^{-1})=\frac{(C_{0}{-C}_{e})\cdot V}{m_{d}}$$

(2) $$R\left(\%\right)=\frac{C_{0}{-C}_{e}}{C_{0}}\cdot 100\%$$

Here, *C_e_* and *C*_0_ are the equilibrium and initial concentration of ReO_4_^−^ ions (mg L^−1^), and *V* is the solution volume (L) applied for the tests.

Based on the results, certain resins were selected for use in the equilibrium studies. In this context, the Langmuir and Freundlich models were applied to describe the adsorption behavior of Re(VII) ions, allowing an evaluation of whether the process follows monolayer ion exchange on homogeneous sites (Langmuir) or exhibits surface heterogeneity (Freundlich). The resins (50 mg of dry mass equivalent) were introduced into 20 mL of Re(VII) solutions containing 30, 60, 125, 250, 500, 1000, and 2000 mg of Re L^−1^ in H_2_O, whose pH was adjusted using 0.1 mol L^−1^ NaOH or 0.1 mol L^−1^ HCl, respectively. The adsorption characteristics were evaluated by applying linear forms of Langmuir (Equation (3)) and Freundlich (Equation (4)) isotherms to the experimental data:

(3) $$\frac{C_{e}}{q_{e}}=\frac{C_{e}}{Q_{0}}+\frac{1}{K_{L}Q_{0}}$$

(4) $$\ln\left(q_{e}\right)=\ln\left(K_{f}\right)+\frac{1}{n}ln(C_{e})$$

Here, *C_e_* is the equilibrium concentration of ReO_4_^−^ ions (mg L^−1^), *q_e_* is the adsorption capacity at equilibrium (mg g^−1^), *Q*_0_ is the maximum adsorption capacity of a given resin (mg g^−1^), and *K_L_* is the binding constant (L mg^−1^). The two terms K*_F_* and 1/*n* are constants related to the adsorption capacity and heterogeneity of the system, respectively.

The separation factor (*R_L_*) was calculated based on the following equation (Equation (5)):

(5) $$R_{L}=\frac{1}{1+{K_{L}C}_{0}}$$

Here, C_0_ is the initial concentration of ReO_4_^−^ ions (mg L^−1^), and K_L_ is the binding constant taken from the Langmuir (Equation (3)) model.

The experimental data regarding Re(VI) adsorption as a function of time were applied to the Lagergren (Equation (6)), pseudo-first-order (PFO), Ho (Equation (7)), pseudo-second-order (PSO), and intra-particle (pore) diffusion (Equation (8)) (IPD) mathematical models, respectively. Kinetic behavior was interpreted by creating kinetic plots according to the above-mentioned models, using Equations (4)–(6) for linear plots [55,56,65,66,67]:

(6) $$\log\left(q_{e}-q_{t}\right)=logq_{e}-\frac{k_{1}}{t}\cdot t$$

(7) $$\frac{t}{q_{e}}=\frac{1}{k_{2}q_{e}^{2}}+\frac{1}{q_{e}}\cdot t$$

(8) qt=kdt12

where *t* (min) is time, q_e_ (mg·g^−1^) is sorption at equilibrium, and q_t_ (mg·g^−1^) is sorption at time t. Parameter k_1_ (min^−1^) was calculated based on the slope of log(q_e_ − q_t_) versus t plot. Parameter k_2_ (g·mmol^−1^·min^−1^) was obtained from the intercept of t/q_e_ against t plot, and parameter k_d_ (mg·g^−1^·min^−1^) was ascertained based on the slope of the q_t_-versus-t^1/2^ plot [66]. The research on adsorption kinetics was carried out using Re(VII) solution in 0.1 mol L^−1^ HCl (1500 mg Re L^−1^), applying the same resin concentration (5 mg mL^−1^) used in the tests on adsorption equilibrium.

### 3.5. Adsorption Selectivity and Re Elution

The selectivity of the anion-exchange resins toward ReO_4_^−^ ions was determined via adsorption tests carried out using multicomponent solutions in 0.1 mol L^−1^ of HCl containing equal molarities (1.4 mmol L^−1^) of Re(VII) and Mo(VI) ions. Furthermore, in the solution V(V), Cu(II) ions were introduced at a concentration level of 4.0 mmol L^−1^. The samples of the anion-exchange resins (equivalent to 100 mg of dry resin) were exposed to 20 mL of multicomponent solutions. These conditions ensured the presence of a deficiency in anion-exchange centers with respect to all the species available for adsorption (10.8 mmol L^−1^of Re, V, Cu, and Mo). The thus-prepared samples of the anion-exchange resins were equilibrated and then separated from the solutions. The concentrations of metals left in the filtrates were then measured using ICP-OES, and Re, V, Cu, and Mo adsorption rates were calculated based on the mass balance. The experimental results were then used to calculate the partition coefficient (K_D_), expressed as the ratio of the amount of metal ions adsorbed by 1 g of a given resin (S, mg g^−1^) to the amount of metal ions remaining in 1 mL of a solution after adsorption (C_e_ 10^−3^) (Equation (9)).

(9) $$K_{D}=\frac{S}{C_{e}}\cdot 10^{3}$$

The KD values calculated for the adsorption of the Re(VII) ions were divided using the KD values obtained for other metals, allowing us to determine the selectivity coefficient toward Re(VII) ions with respect to V(V), Cu(II), and Mo(VI) (Equation (10)):

(10) $$s_{i}=\frac{K_{Re}}{K_{Mo,V,Cu}}$$

Finally, the anion-exchange resins that had separated from multicomponent solutions after the adsorption test were placed in contact with NaOH solution (1 mol L^−1^) to desorb the metals adsorbed. After equilibration, the concentrations of Re, V, Cu, and Mo were determined, and, based on the mass balance, the elution (%) for each metal was estimated.

Similarly, we performed cyclic adsorption and elution of Re(VII) over four cycles. For the test, 100 mg of a resin was placed in contact with 20 mL of a single-component solution containing 500 mg of Re L^−1^ in 0.1 mol L^−1^ of HCl. After 24 h, the polymer was separated via filtration, washed with water, and then introduced to 20 mL of 0.1 mol L^−1^ of NaOH. The collected solutions were subjected to ICP-OES analysis, and the concentrations of Re obtained were used to make a mass balance-based estimate of adsorption–desorption efficiency over the tested cycles.

### 3.6. Flow Mode Adsorption of Re

The flow-mode adsorption process of Re adsorption was carried out by packing a polymer sample into a self-designed and self-produced ion-exchange column. The column was modeled using Autodesk Inventor Professional 2025 (licensed for educational use by the Wroclaw University of Science and Technology). The resulting 3D geometry was exported in .stl format and further processed using PreForm 3.41.0 software (Formlabs, Sommerville, MA, USA) to prepare the file for additive manufacturing. Fabrication was carried out via stereolithography using a Formlabs Form 3 (Formlabs, Sommerville, MA, USA) printer, utilizing Clear v4 resin as the printing material. Post-processing involved rinsing the printed column in isopropanol using the FormWash system (Formlabs, Sommerville, MA, USA), followed by UV curing at 60 °C in a FormCure (Formlabs, Sommerville, MA, USA) unit to complete polymerization. The resultant column had the following dimensions: 150 mm in length and 5 mm in diameter. The ion-exchange column was loaded with a polymer sample, whose bed volume (BV) was 1.5 mL. Furthermore, 3D-printed thread joints were used to seal the system.

Then, the column with resin was connected to a peristaltic pump (Masterflex MFLX07522-30-EU, Masterflex SE, Gelsenkirchen, Germany) and a Büchi B-684 (Büchi Labortechnik AG, Flawil, Switzerland) fraction collector. The system set in this way was fed in the counter-flow direction using a solution containing 100 mg of Re L^−1^ in 0.1 mol L^−1^ of HCl at a flow rate of 60 BVs h^−1^. The outflows were collected, and the Re concentration was determined using ICP-OES. The gathered data were then used to create a breakthrough curve as a C_e_/C_0_ = f(BVs) function, where C_e_ and C_0_ are the equilibrium and initial Re concentrations, respectively, and BVs denotes resin bed volumes.

## 4. Conclusions and Future Perspectives

In this study, we successfully developed and characterized novel anion-exchange resins modified with weakly basic amine groups for the selective recovery of Re(VII) ions from simulated by-products of Cu-Mo ore processing. The synthesized resins demonstrated high adsorption capacities, with maximum values ranging from 390.5 to 435.4 mg Re g^−1^ and favorable selectivity for monovalent ReO_4_^−^ ions over competing ions such as MoO_4_^2−^, Cu^2+^, V^3+^, V^2+^, (VO_2_)^+^, and (VO)_2_^+^ existing at pH 1 [47]. Among the tested resins, the PIP and BAPA resins exhibited the highest selectivity and elution efficiencies, while the HEP resin displayed versatility across a wide pH range, though its stripping efficiency was probably affected by the potential reduction of the Re(VII) ions during their adsorption.

The adsorption equilibrium followed the Langmuir isotherm model, confirming the chemical ion-exchange mechanism, while the selectivity indices highlighted the resins’ preference for the ReO_4_^−^ ions in the multi-metallic solutions. Furthermore, complete desorption of Re from the PIP and BAPA resins was achieved, showcasing their suitability for practical applications in cyclic adsorption–desorption processes. These findings suggest that the resins can be effectively employed for Re recovery under acidic conditions, which are typical for industrial waste streams.

This work contributes to the enhancement of resource efficiency and promotes environmental sustainability by offering an innovative solution for recovering Re from secondary sources. The proposed materials not only address the growing demand for this critical metal but also offer promising potential for integration into existing technologies for the recovery of valuable resources from industrial by-products, thereby supporting a circular economy. While the tested systems simulate key components of Cu-Mo ore-processing intermediates, real-world solutions may present additional challenges, such as fouling from organic matter, resin degradation under prolonged acidic exposure, and interference from other anionic species not included in this study. In this context, future work could evaluate the long-term stability of the synthesized resins, as well as their performance in complex matrices and potential scaling-up, to fully assess their industrial viability. Although a detailed cost analysis was beyond the scope of this study, materials and procedures analogous to those used for commercial ion exchangers were used in the synthesis of the resins, suggesting that implementation into existing Re recovery processes could be economically feasible. Finally, we recommend conducting further studies employing molecular modeling and theoretical simulations to better understand the specific interactions between ReO_4_^−^ and weakly basic amine functionalities and provide mechanistic insights into the selectivity observed.

## Figures and Tables

**Table 1:** The elemental composition, water regain, and anion-exchange capacity of the anion-exchange resins.

Sample	Elemental Composition (wt%)				W ^a^ (RSD, %)	Z_H_^b^	
	C	H	N	S	O		
BAPA	65.36 ± 0.05	7.26 ± 0.03	5.08 ± 0.04		-	0.18 (0.16)	2.14
PIP	68.25 ± 0.06	6.58 ± 0.06	10.25 ± 0.08	-	-	0.10 (0.00)	2.18
TSC	69.90 ± 0.03	5.70 ± 0.04	7.28 ± 0.02	10.88 ± 0.04	-	0.13 (3.77)	0.44
AHP	72.99 ± 0.03	6.25 ± 0.04	3.45 ± 0.03	-	8.66 ± 0.07	0.22 (0.70)	0.88
HEP	63.42 ± 0.16	7.30 ± 0.01	5.72 ± 0.04	-	11.68 ± 0.13	0.08 (1.06)	1.74
AHPI	76.28 ± 0.09	6.26 ± 0.04	0.37 ± 0.01	-	4.37 ± 0.12	0.14 (1.37)	0.00
TA	70.32 ± 0.04	6.08 ± 0.03	3.59 ± 0.17	4.18 ± 0.03	-	0.18 (0.38)	0.00

^a^ Water regain (g g^−1^). ^b^ Hecker’s anion-exchange capacity (mmol g^−1^).

**Table 2:** The Langmuir (Equation (3)) Re(VII) ion adsorption parameters calculated based on the experimental points.

Resin	Q_0_ ^a^	Q_0m_ ^b^	K_L_ ^c^	R_L_ ^d^	R^2^
BAPA	435.4	2.3	0.0023	0.4987	0.9974
PIP	419.2	2.3	0.0024	0.4993	0.9986
HEP	390.5	2.1	0.0026	0.4978	0.9956

^a^ Langmuir maximum adsorption capacity (mg g^−1^). ^b^ Langmuir maximum adsorption capacity (mmol g^−1^). ^c^ Binding constant [L mg^−1^]. ^d^ Separation factor. R^2^ Correlation coefficient.

**Table 3:** The selectivity indices of Re adsorption with respect to Mo, V, and Cu.

	Re(VII) Selectivity Index		
Resin	vs. Mo(VI)	vs. V(V)	vs. Cu(II)
BAPA	2.9	→∞	
PIP	1.9	44	103
HEP	2.4	116	438

**Table 4:** Comparison of recent anion-exchange resins in terms of Re adsorption.

Resin	Re adsorption (mg Re g^−1^)	Selectivity (Re over Mo)	Regeneration Efficiency	Notes	Reference
Purolite A170	~100	Reduced in Re-Mo systems	High; efficient with ammonia (based on prior research [60])	Commercially available; performance affected by competing Mo ions.	[4]
Modified D201	92.16	High; Re: 97.46%, Mo: 0.38%	~95%	Excellent selectivity in acidic solutions; stable over multiple cycles.	[35]
ZS15 Weak-Base Resin	Not specified	High; effective in Re-Mo systems	Efficient desorption with low-concentration ammonia	Suitable for industrial applications; good selectivity and regeneration.	[34]
D990 Resin	162	Not specified	97.3% with 6% ammonia	Strong adsorption from copper smelting solutions; effective regeneration.	[59]
PS-G-4VP-IE Resin	252	High over pH 1.5–6	Not specified	High capacity across a wide pH range; suitable for various conditions.	[61]
VBC-co-DVB/1-(3-aminopropyl)imidazole	303	Selectivity index: 220	Not specified	Resin synthesized entirely in a microwave radiation.	[41]
VBC-co-DVB/ bis(3-aminopropyl)amine VBC-co-DVB 1-(2-pyrimidinyl)piperazine	435 419	Selectivity index: 2.9 Selectivity index: 1.9	~100% Re elution	Maintained activity over 4 sorption/desorption cycles High processing ability in flow mode.	This work

## Data Availability

The data are stored under the permanent identifier https://doi.org/10.18150/4OVWUB.

## Supplementary Materials

The following supporting information can be downloaded at https://www.mdpi.com/article/10.3390/ijms26157563/s1.
