# Supplementary material for: Novel Anion-Exchange Resins for the Effective Recovery of Re(VII) from Simulated By-Products of Cu-Mo Ore Processing
Source: Int J Mol Sci. 2025 Aug 5;26(15):7563. doi: 10.3390/ijms26157563 (PMC12347548; doi:10.3390/ijms26157563)
Supplement: Supplementary file 1 [file ijms-26-07563-s001.zip › ijms-3727698-supplementary.pdf]

Supplementary Materials to

Novel Anion Exchange Resins for the Effective Recovery of Re(VII) from Simulated by-Products of Cu-Mo Ore Processing

Piotr Cyganowski[1*], Pawel Pohl[2], Szymon Pawlik[1], Dorota Jermakowicz-Bartkowiak[1]

[1]*Department of Process Engineering and Technology of Polymer and Carbon Materials, Wroclaw University of Science and Technology, 27 Wybrzeze St. Wyspianskiego, 50-370 Wroclaw, Poland*

[2]*Department of Analytical Chemistry and Chemical Metallurgy, Wroclaw University of Science and Technology, 27 Wybrzeze St. Wyspianskiego, 50-370 Wroclaw, Poland*

*Corresponding Author: prof. Piotr Cyganowski: Phone +48 58-523-52-08, e-mail address: piotr.cyganowski@pwr.edu.pl

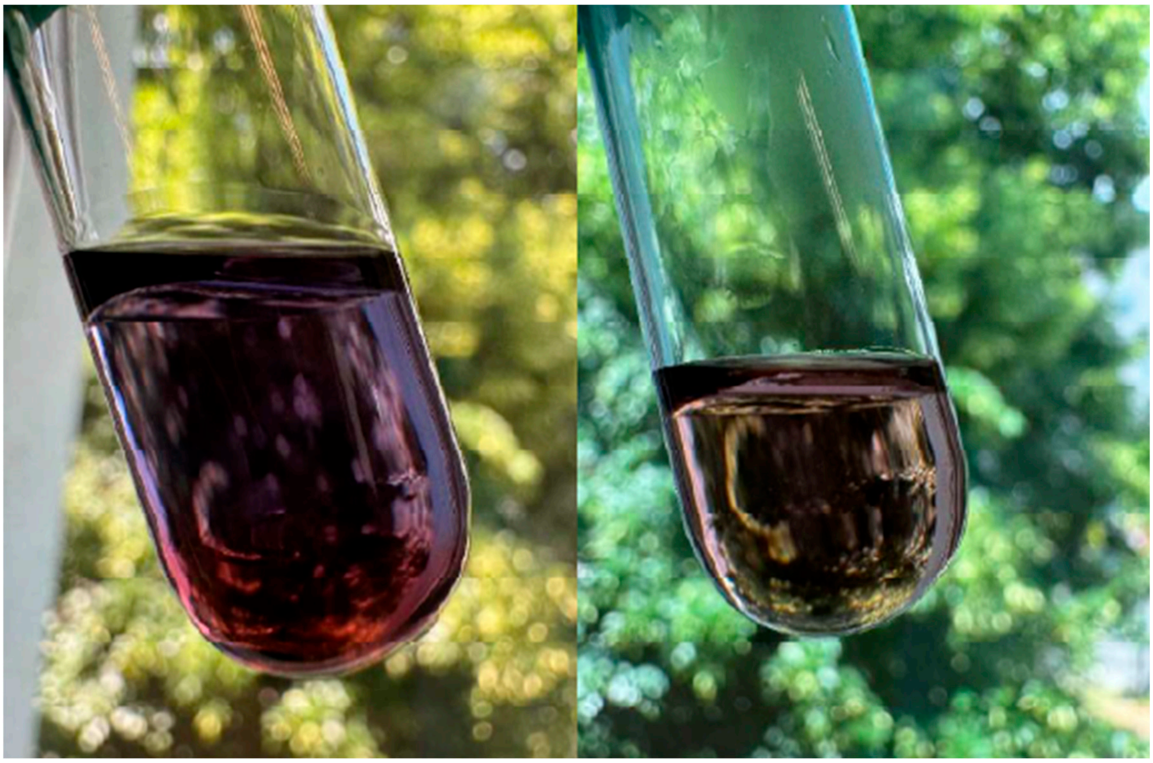

Figure S1. A photograph of BAPA sample subjected to the colorimetric test carried out in the presence of ninhydrin. The purple color is the evidence of $–NH_2$ groups on the polymer's surface

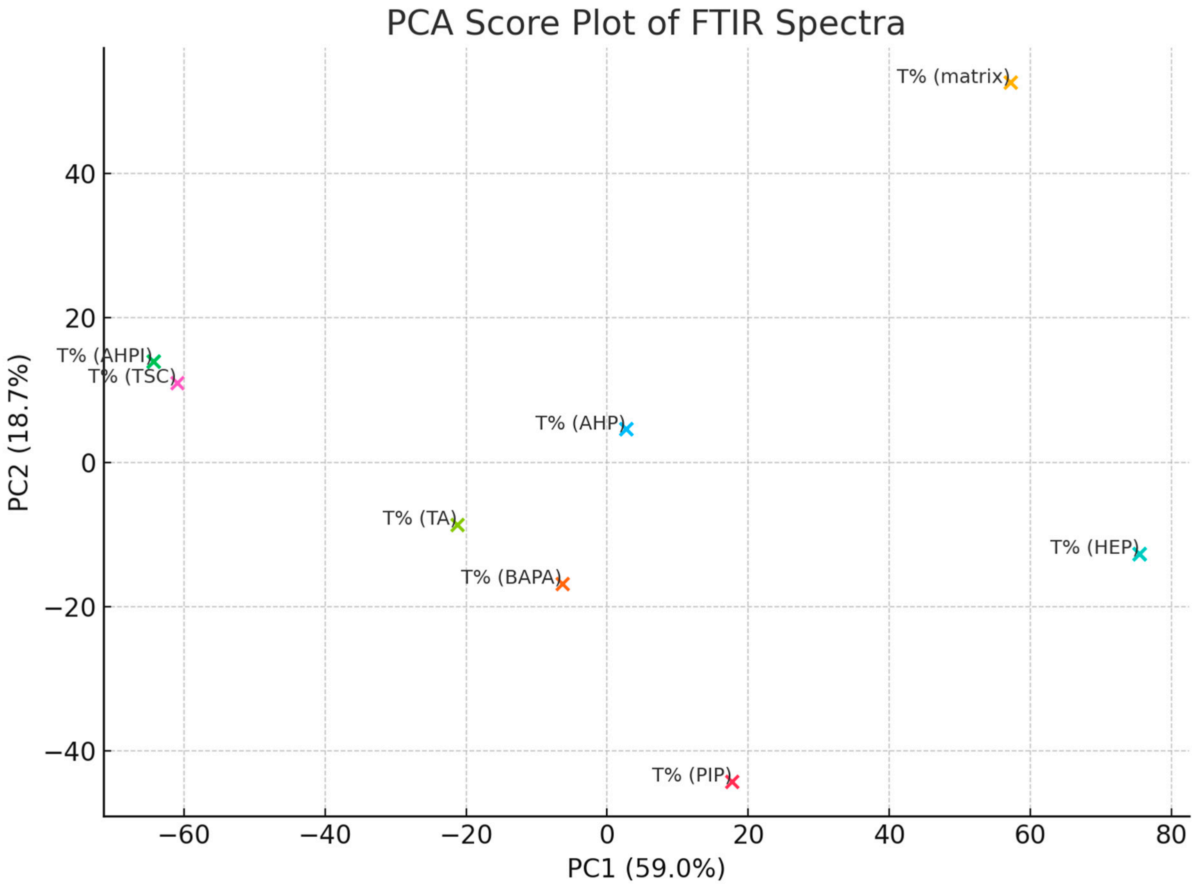

Figure S2. Principal Component Analysis (PCA) score plot showing the distribution of polymer samples based on their FTIR spectra in the 4000–400 cm^{-1} range. The analysis was performed using a standard PCA algorithm implemented with the scikit-learn library in Python, where each spectrum was mean-centered prior to dimensionality reduction. The plot illustrates separation trends between the base copolymer and the amine-functionalized resins, primarily along PC1 and PC2, which together explain the majority of spectral variance. The Python algorithm was generated with the aid of ChatGPT-4o model.

Table S1. SEM/EDX analysis of VBC-co-DVB copolymer, and BAPA, PIP, HEP anion exchange resins

**Sample**	**EDX surface composition (wt%)**				
	C	N	O	Cl	Re
VBC-co-DVB copolymer	65.52	5.35	5.57	23.56	-
BAPA	55.15	10.79	10.67	13.19	10.21
PIP	50.39	15.56	11.07	8.62	14.35
HEP	69.32	14.26	7.80	8.62	undetected

Comment on experimental uncertainties and error analysis

Due to the synthetic nature of this study, in which anion exchange resins were synthesized from monomers via multistep chemical modifications, the total amount of each resin available for analysis was strictly limited to several grams. As a result, extensive replicate experiments, particularly those requiring larger sample quantities (e.g., ion-exchange capacity measurements, isotherm studies), could not be conducted for all procedures. Reproducibility assessments were therefore prioritized for key parameters, including elemental analysis of functional groups and adsorption studies.

Where feasible, replicate measurements were carried out and standard deviations were calculated and included in Figures 4 and 5 and Table 1. Importantly, the adsorption values obtained (typically in the range of 300–435 mg Re/g) greatly exceed the expected magnitude of instrumental error from concentration measurements (typically <5%), rendering the absolute error bars small in relative terms.

Further, based on the technical aspects of the carried out research, in which all of the values originate from mass balances, we identify concentration measurements as a major factor that could contribute to the potential errors. These however were comparable to those encountered in similar resin development studies. For the reader's guidance please refer to raw data contained in a public repository (https://doi.org/10.18150/4OVWUB), where all results from the analyses with prepared repetitions are disclosed.

Nonetheless, the authors acknowledge that larger-scale studies and more comprehensive error propagation would be valuable in future work and industrial validation stages.

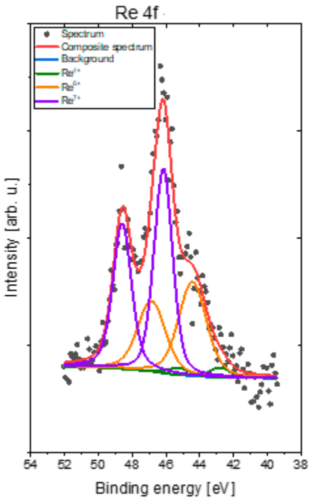

Figure S3. Re 4f XPS spectrum of the HEP sample after adsorption of Re(VII). The spectrum is a part of results previously presented supplementary materials to Cyganowski et al. Scientific Reports 13.1 (2023): 12789 published under CC BY creative commons license
